# Diurnal modulation of subthalamic beta oscillatory power in Parkinson’s disease patients during deep brain stimulation

**DOI:** 10.1038/s41531-022-00350-7

**Published:** 2022-07-08

**Authors:** Joram J. van Rheede, Lucia K. Feldmann, Johannes L. Busch, John E. Fleming, Varvara Mathiopoulou, Timothy Denison, Andrew Sharott, Andrea A. Kühn

**Affiliations:** 1 Medical Research Council Brain Network Dynamics Unit, Nuffield Department of Clinical Neurosciences, University of Oxford, Mansfield Road, Oxford, OX1 3TH United Kingdom; 2 Department of Neurology, Charité - Universitätsmedizin Berlin, corporate member of Freie Universität Berlin and Humboldt-Universität zu Berlin, Berlin, Germany; 3 Berlin Institute of Health at Charité Universitätsmedizin Berlin, Berlin, Germany; 4 Institute of Biomedical Engineering, Department of Engineering Science, University of Oxford, Oxford, United Kingdom; 5 Berlin School of Mind and Brain, Charité Universitätsmedizin Medicine, Berlin, Germany; 6 NeuroCure Clinical Research Centre, Charité Universitätsmedizin, Berlin, Germany

**Keywords:** Parkinson's disease, Parkinson's disease, Circadian rhythms and sleep, Parkinson's disease

## Abstract

Beta-band activity in the subthalamic local field potential (LFP) is correlated with Parkinson’s disease (PD) symptom severity and is the therapeutic target of deep brain stimulation (DBS). While beta fluctuations in PD patients are well characterized on shorter timescales, it is not known how beta activity evolves around the diurnal cycle, outside a clinical setting. Here, we obtained chronic recordings (34 ± 13 days) of subthalamic beta power in PD patients implanted with the Percept DBS device during high-frequency DBS and analysed their diurnal properties as well as sensitivity to artifacts. Time of day explained 41 ± 9% of the variance in beta power (*p* < 0.001 in all patients), with increased beta during the day and reduced beta at night. Certain movements affected LFP quality, which may have contributed to diurnal patterns in some patients. Future DBS algorithms may benefit from taking such diurnal and artifactual fluctuations in beta power into account.

## Introduction

Subthalamic deep brain stimulation (DBS) is a highly effective therapy for Parkinson’s disease (PD)^1,2^. In addition to its clinical utility, DBS has provided unique insight into the pathophysiology of movement disorders, leading to the concept of abnormal network activity in brain disorders^3–5^. The best characterized pathophysiological activity is an abnormal increase in the power and stability of beta oscillations in the neuronal activity of the subthalamic nucleus (STN) in the hypodopaminergic state^3,6^. Reduction of these pathologically enhanced beta oscillations correlates with the efficacy of treatment of akinetic/rigid symptoms using dopamine replacement or continuous, high-frequency (130 Hz) DBS^7–9^. In the subthalamic local field potential (LFP), beta oscillations occur in bursts of high amplitude activity, the duration of which also relates to symptom severity^10^.

These observations have inspired closed-loop, adaptive DBS (aDBS), where the amplitude of local beta-band activity is used to adjust stimulation in response to pathophysiological activity^11–15^. This approach has recently become more feasible through implementation on new DBS devices with the capacity to monitor beta power during continuous stimulation^9,16–20^. aDBS utilizes changes in beta amplitude on the scale of seconds and minutes to control stimulation. However, in addition to the influence of stimulation amplitude and medication, beta power is likely to fluctuate in relation to processes on longer timescales. Most notably, polysomnographic studies in PD patients point towards markedly decreased beta amplitude during several stages of sleep^21^. In recordings from DBS leads in the STN of PD patients during off-stimulation periods, it has been found that beta power is greatly reduced during non-rapid-eye-movement sleep (NREM) epochs compared to waking epochs^20,22,23^. To what extent such differences in beta power between sleep and waking hours are still observed in PD patients undergoing clinically efficacious high-frequency DBS is less clear (though a reduction in beta during sleep was noted in a recent paper trialing a new aDBS algorithm^24^). In the aforementioned studies, epochs of beta power were either selected from a single sleep-wake cycle^22,23^ or collapsed across sleeping and waking epochs from multiple recording days^20,24^, and no distinctions were made between waking epochs at different times of the day. It is therefore not known how beta power evolves over the full 24-h diurnal cycle, whether an individual patient’s beta power profile is consistent across days on longer timescales (weeks/months), or whether there are consistent diurnal variations across patients other than those driven by the sleep/wake cycle. It is also not clear how previously observed differences between sleeping and waking will be impacted by continuous DBS. These issues are important for the future development and use of closed-loop devices.

Focusing on the 24-h diurnal cycle is relevant, as many neural processes other than the sleep-wake cycle are modulated by the output of the suprachiasmatic nucleus of the hypothalamus, which provides the brain’s principal circadian clock^25,26^. This intrinsic circadian rhythm generator could influence beta oscillations through the vast number of (neuro)physiological processes that are influenced by changes in its output throughout the entire 24-h cycle^27,28^. Finally, levels of physical activity are also related to the time of day. This may lead to physiological modulation of beta in response to movements^29–33^, but may also result in temporal biases in potential artifact sources. It is already known that the heartbeat can affect measures of beta power in certain DBS devices^34,35^, and movements themselves present another potential source of transient artifacts in LFP recordings^36,37^. As the parameters for aDBS are currently set under controlled conditions during clinician working hours, both physiological and artifactual diurnal changes in beta measurement could impact aDBS effectiveness outside of the clinic.

Here we analysed long-term (18-59 days) recordings of STN LFP beta-band power during continuous DBS from PD patients implanted with the Medtronic Percept DBS device, and show that beta amplitude can be significantly modulated according to time of day. We provide evidence that such fluctuations can be driven by physiological processes in some patients, but by movement-related artifacts in others. These findings highlight the importance of understanding and interpreting the sources of slow fluctuations in beta amplitude recorded from chronic sensing devices, for delivery of aDBS and for scientific investigation.

## Results

### Measured STN beta power fluctuates in a 24-h cycle and is reduced during the night

**Fig. 1:**
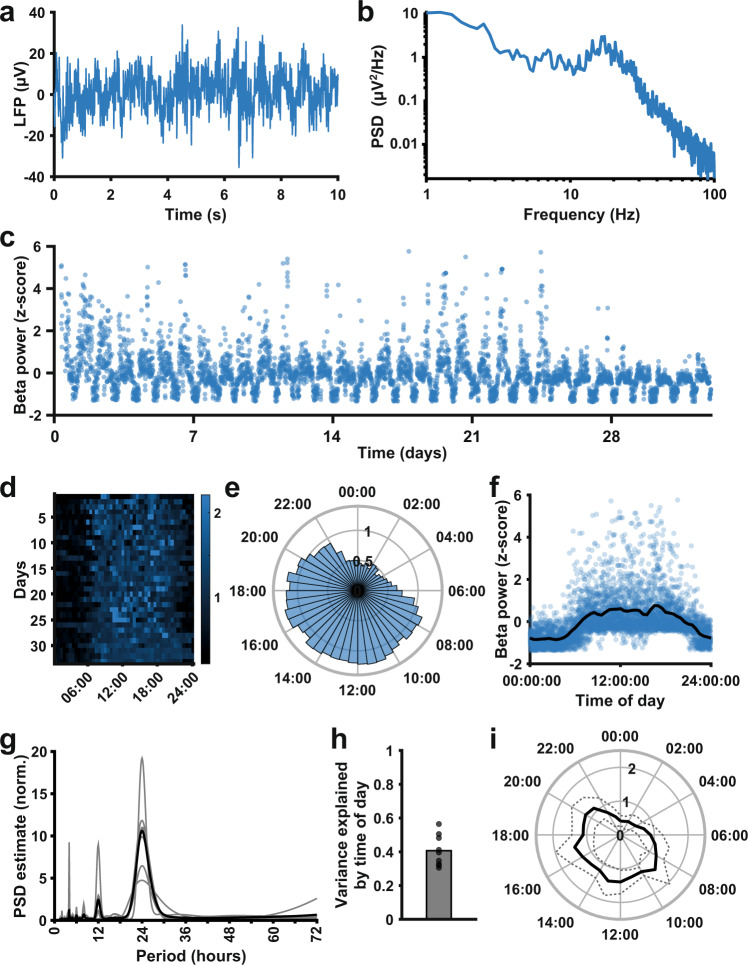
Beta oscillatory power measured with the Percept DBS device shows a consistent diurnal pattern with decreased beta during the night. **a** A 10-s LFP recording from the STN of a Parkinson’s disease patient implanted with the Medtronic Percept DBS device, acquired during the BrainSense signal test. **b** Power spectrum of the LFP segment in **a** (obtained using Welch’s method) presented on a log-log scale, showing a peak in the beta frequency range. **c** Mean STN peak-to-peak beta power (µVp, sampled every 10 min) over a 1-month period, from the same example STN (after outlier removal). **d** Heat map of beta power (detrended by normalizing each day to its median value) across the 24 h of the day for all days in the data collection period, for the same example STN. **e** Detrended beta power across the 24-h diurnal cycle generated from the data in **d**. For each day, the median beta power was calculated for each 30-min time bin, and the bar height in the circular bar graph represents the median across days. **f** Normalized beta power measurements plotted against the time of day the measurements were taken for the same example STN, showing a consistent daily pattern of beta power. The black line represents a linear fit through the mean beta power for the time of day (means obtained by dividing the day into 30-min time bins). **g** Periodogram of the beta power signal for all STN (normalized; estimated using Welch’s method). The population mean (black) highlights a clear peak at a period of 24 h, which is present in all individual beta power time series (gray lines, *n* = 9). **h** The proportion of variance explained by time of day, estimated using fits such as illustrated in **d**, for all STN in the data set (mean 0.41 ± 0.092; *n* = 9; *p* < 0.001 for all-time series, temporal shuffling test). **i** Mean (solid line) and standard deviation (dashed line) of detrended beta power around the diurnal cycle across all STN time series (*n* = 9).

In six patients implanted with bilateral STN DBS electrodes, we acquired measurements of beta oscillatory power of the LFP every 10 min. An LFP stream, power spectrum, and long-term beta power measurements are shown for a single STN in Fig. 1a–c. Alternating periods of increased and reduced beta power are clearly visible in the long-term measurements. Specifically, beta power was consistently greater during the day and reduced during the night (Fig. 1d–f). Indeed, all STN time series showed a clear 24 h periodicity (Fig. 1g), suggesting that the diurnal cycle had a strong effect on beta power. Time of day fits (such as in Fig. 1e) explained a significant proportion of the variance in beta power in all-time series (Fig. 1h; mean 0.41 ± 0.092; *p* < 0.001 for all-time series, temporal shuffling test). Moreover, all STN LFPs showed a pattern of increased beta power during the day and decreased beta power during the night (Fig. 1i), though there was individual variability in diurnal beta profiles (see Supplementary Figs. 1–6).

### Transitions between sleeping and waking periods are associated with beta fluctuations

**Fig. 2:**
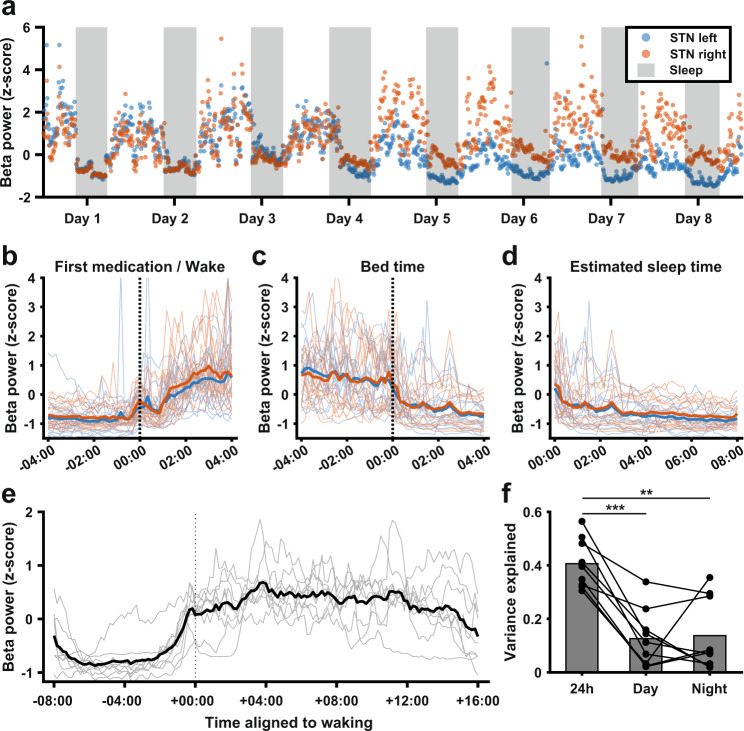
Diurnal variations in beta power are largely accounted for by transitions between sleeping and waking periods. **a** Normalized beta power in the left and right STN (blue and orange, respectively) of one patient over a period of 8 days during which the patient recorded when they went to bed and when they rose in the morning. Gray areas represent the time the patient was in bed for the night. **b** Normalized beta power as in **a** for the full 21-day recording period, aligned to the time of waking. Blue and orange lines represent the mean for the left and right STN respectively, while thin blue and orange lines represent the corresponding individual time series. **c** Normalized beta power as in **a** for the full 21-day recording period, aligned to the time of going to bed. Blue and orange thick lines represent the mean for the left and right STN respectively, while thin blue and orange lines represent the corresponding individual time series. **d** Normalized beta power as in **a** for the full 21-day recording period, between going to bed and waking up. Blue and orange thick lines represent the mean for the left and right STN respectively, while thin blue and orange lines represent the corresponding individual time series. **e** Average beta power aligned to estimated average wake-up time for all STN time series in the data set (gray lines, *n* = 9) and mean across all-time series (thick black line). **f** Variance explained by time of day across the whole 24 h cycle vs. during the day or night alone. Full 24 h: 0.41 ± 0.092; Day only: 0.13 ± 0.11, Wilcoxon’s signed-rank = 45, *p* = 0.039 vs 24 h; Night only: 0.14 ± 0.13, Wilcoxon’s signed-rank = 43, *p* = 0.012 vs. 24 h; *n* = 9 STN time series.

To examine whether the diurnal profile of beta power observed was driven by periods of sleeping and waking, we acquired additional bilateral STN data from one patient (#5), who was asked to log their bedtime and rise time each day for a total period of 21 days (Fig. 2a). Clear transitions in beta power were present around the times of going to bed and waking up this patient (Fig. 2a–c), and during estimated sleeping hours beta power was consistently reduced (Fig. 2d). Consistent with this, there was a sharp increase in beta power slightly before their first scheduled medication time across patients (Fig. 2e), and compared to the variance explained by time of day across the full diurnal cycle, time of day accounted for much less variance during day- or night-only epochs (Fig. 2f; full 24 h: 0.41 ± 0.092; day only 0.13 ± 0.11, Wilcoxon’s signed-rank = 45, *p* = 0.039 vs 24 h; night only: 0.14 ± 0.13, Wilcoxon’s signed-rank = 43, *p* = 0.012 vs. 24 h; *n* = 9 STN time series). Nevertheless, for most patients, the variance explained by time of day was still significant during day epochs only (*p* < 0.001 in 7/9 patients, temporal shuffling test) and night epochs only (*p* < 0.001 in 6/9 patients and *p* < 0.05 in 1/9 patients, temporal shuffling test), indicating remaining systematic beta fluctuations not explained by the sleep-wake cycle.

### Frequency-specificity of diurnal LFP power modulation

**Fig. 3:**
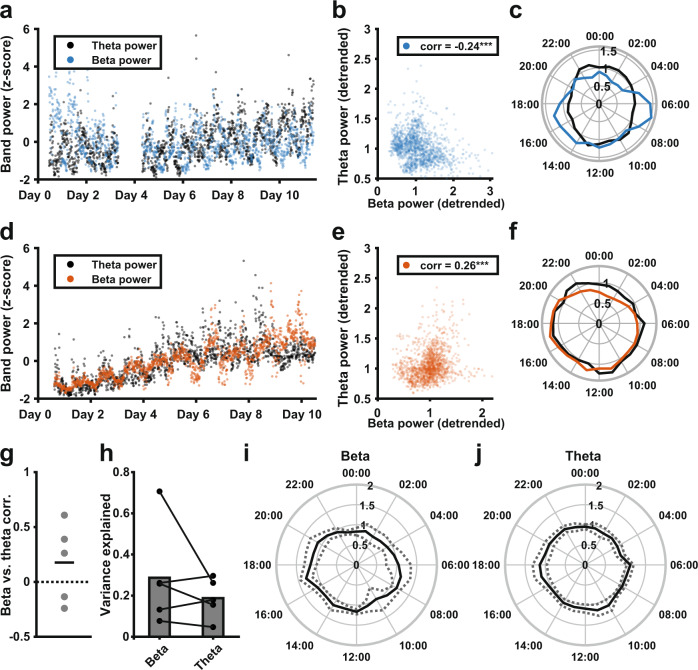
Correlations in long-term measurements of neural oscillatory power across frequency bands. **a** Normalized beta power (blue) and theta power (black) were collected concurrently from the left and right STN of one example patient. **b** Scatter plot of detrended beta power vs detrended theta power shown in **a** (data were detrended by normalizing values for each day to that day’s mean). For this patient, there was a negative correlation between beta and theta (*r* = -0.24, *p* < 0.001). **c** Median detrended beta (blue) and theta (black) power across the 24-h diurnal cycle, showing different diurnal profiles of theta and beta in this example patient. **d** Normalized beta power (orange) and theta power (black) were collected concurrently from the left and right STN of a second example patient. **e** Scatter plot of detrended beta power vs detrended theta power shown in **d** (data were detrended by normalizing values for each day to that day’s mean). For this patient, there was a positive correlation between beta and theta (*r* = 0.26, *p* < 0.001). **f** Median beta (orange) and theta (black) power across the 24-h diurnal cycle, showing the overlap in the diurnal profiles of beta and theta power in this second example patient. **g** Pearson’s correlation coefficients for concurrently collected contralateral STN beta and theta power measurements (detrended; 0.18 ± 0.36, *n* = 5). **h** Variance explained by time of day in concurrently collected beta and theta power measurements (*t*(4)=1.1, *p* = 0.33, 5% confidence intervals (−0.15, 0.35), paired *t*-test, *n* = 5). **i**, **j** Mean (solid black) and standard deviation (dashed, gray) concurrent diurnal beta (**i**) and theta (**j**) power profiles around the diurnal cycle, across the STN of five patients.

As the chronically streamed data represents the mean power in a single frequency band, broadband fluctuations in LFP power are indistinguishable from fluctuations specific to the target frequency. To investigate whether the diurnal fluctuations we observed were specific to the beta band, we compared concurrently collected beta and contralateral theta power in five patients. If diurnal changes are driven by broadband artifacts affecting both hemispheres, these signals should be highly correlated. Figure 3a, d show beta and theta band power for two patients (plotted against each other in Fig. 3b, e), illustrating that beta and contralateral theta power could be either positively or negatively correlated. The beta and theta time series for all patients in the dual-band data set are provided in Supplementary Fig. 7. Overall, correlation coefficients between beta and theta power ranged from −0.24 to 0.61 (mean 0.18 ± 0.36, *n* = 5). Beta and theta power could also show different diurnal profiles (Fig. 3c, f). In the beta signal, the proportion of variance explained by time of day was 0.29 ± 0.25, while for the theta signal this was 0.19 ± 0.10. While this difference was not significant (*t*(4) = 1.1, *p* = 0.33, 5% confidence intervals (−0.15,0.35), paired *t*-test), it demonstrates for individual cases that the diurnal patterns cannot be fully explained by broadband signal power. Indeed, across the patient population, beta band power was less uniformly distributed around the diurnal cycle than theta band power (Fig. 3i, j).

### Artifacts generated by everyday movements influence measures of LFP band power acquired with the Percept DBS device

**Fig. 4:**
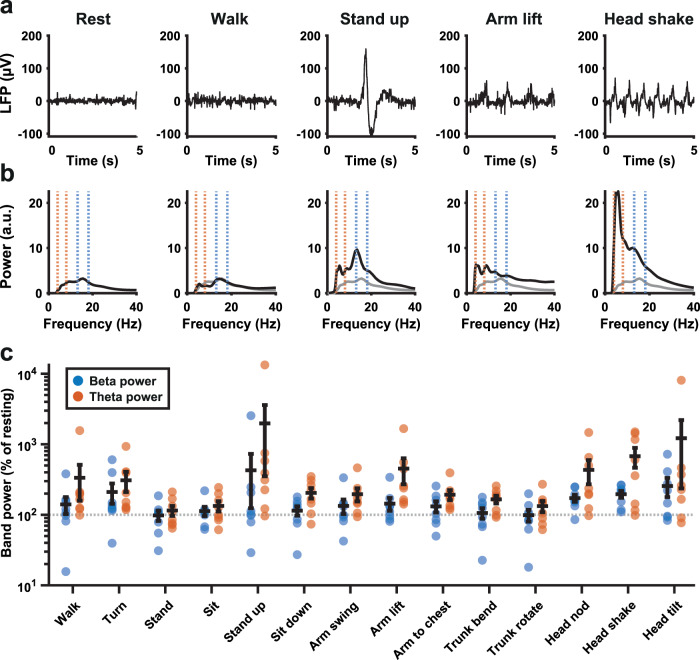
Common movements and their impact on measures of beta and theta band power. **a** Five-second LFP recordings from the STN of one patient collected with DBS on while they were resting, walking, standing up from a sitting position, making a winging motion with the arms, or shaking the head after application of a low-pass filter at <98 Hz and high-pass filter at >4 Hz. **b** Global wavelet power spectra (arbitrary units) corresponding to the LFP collected during the events depicted in **a**. Blue dotted lines indicate the beta peak window for this STN and orange dotted lines indicate the theta band. The resting spectrum on the left is replicated as a gray line across the other spectra for comparison. **c** Beta (blue) and theta (orange) band power measures as a percentage of resting power, from LFPs collected during a series of different movements (see Table 2 for all absolute and relative values; *n* = 8 STN LFPs from four patients). The beta band was defined as 5 Hz around the STN-specific beta peak, while the theta band was 4–8 Hz for all patients.

The presence of correlations across frequency bands in several patients warranted further investigation of what might underlie diurnal broadband influences on the beta power measure. Movement artifacts could present a confound, as they would affect a large part of the power spectrum and would be more numerous during waking hours compared to during the night. We therefore asked a subset of patients to carry out a range of movements in the clinic while acquiring a bilateral STN LFP stream. Individual movements could have a clear impact on the LFP and the associated power spectrum (Fig. 4a, b), and many movements increased both beta and theta power estimates (Fig. 4c and Table 1). Particularly notable was a consistent effect of common head movements for all patients. Theta band power was increased even more than beta during most movements, with average increases of up to an order of magnitude.

**Table 1:** Common movements and their impact on measured beta and theta power.

Activity	Beta power (µVp)	Relative beta (%)	Theta power (µVp)	Relative theta (%)
Rest	3.76 ± 3.82	100 ± 0	3.31 ± 2.20	100 ± 0
Walk	3.39 ± 2.11	140 ± 107	8.18 ± 8.33	334 ± 497
Turn	4.45 ± 2.49	210 ± 189	8.04 ± 6.89	308 ± 278
Stand	3.08 ± 2.42	98 ± 46	3.88 ± 3.17	114 ± 52
Sit	3.81 ± 3.17	112 ± 48	4.57 ± 3.77	133 ± 63
Stand up	6.90 ± 8.79	427 ± 858	25.37 ± 36.89	1976 ± 4595
Sit down	3.17 ± 2.35	114 ± 48	6.61 ± 5.08	204 ± 98
Arm swing	3.67 ± 2.35	133 ± 88	5.91 ± 4.00	196 ± 117
Arm lift	4.81 ± 4.05	143 ± 84	17.73 ± 29.24	452 ± 512
Arms to chest	3.91 ± 2.79	131 ± 67	6.83 ± 6.49	192 ± 88
Trunk bend	2.89 ± 1.78	105 ± 50	5.94 ± 5.01	165 ± 59
Trunk rotate	2.59 ± 1.68	98 ± 53	5.05 ± 4.97	132 ± 67
Head nod	7.51 ± 9.75	172 ± 55	13.35 ± 12.14	433 ± 460
Head shake	7.52 ± 7.92	196 ± 61	22.18 ± 24.41	676 ± 600
Head tilt	15.53 ± 29.83	255 ± 219	60.67 ± 150.2	1218 ± 2779

(*n* = 8 STN LFPs from four patients).

**Fig. 5:**
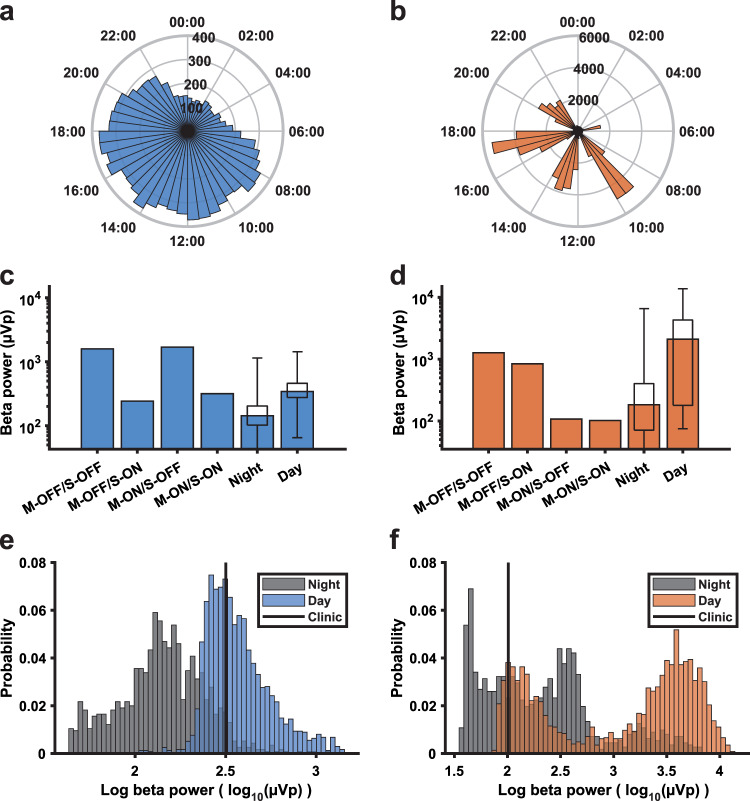
A comparison of beta power measures collected in-clinic and during long-term data collection. **a**, **b** Diurnal profile of beta power across the 24 h cycle from two STN leads from different patients. Each bar represents the median beta power (µVp) in the time bin across all days. **c**, **d** In-clinic measures of beta power collected with DBS on and off (S-ON/S-OFF), while on and off medication (M-ON/M-OFF), compared to the median and distribution of beta power values (boxplots, the box indicates the 25th–75th percentile range, whiskers show the full range of the data) obtained from long-term data collection during the night-time (00:00–06:00) and daytime (08:00–20:00), for the same two examples. **e**, **f** Distributions of daytime (08:00–20:00) and night-time (00:00–06:00) log-transformed beta power measurements (log_10_(µVp)) during the long-term sensing period for the same examples. The black line indicates the in-clinic measurement taken while on dopaminergic medication during DBS on.

To illustrate to what extent long-term data might be affected by movement artifacts, we collected in-clinic daytime measures on and off anti-parkinsonian medication and on and off DBS for two STN leads with different diurnal LFP profiles (Fig. 5a, b). One STN showed a smooth diurnal profile across the 24-h cycle (Fig. 5a) while the other STN showed extreme intra-day peaks (Fig. 5b). This second patient had strong medication-related dyskinesia resulting in vigorous involuntary movement at certain times during the day. For the first STN, the measure collected in-clinic (on medication and with DBS) lies in the center of the distribution of daytime long-term beta measurements, and to the right of most night-time measurements (Fig. 5c, e). However, for the second STN, the in-clinic measure was much lower than most of the long-term daytime and even many night-time measurements. Moreover, the long-term data for daytime has a strongly bimodal distribution (Fig. 5d, f), consistent with the presence of dyskinesia-related movement artifacts. Day- and night-time distributions of beta power are provided for all patients in Supplementary Figs. 1–6g, n.

## Discussion

Using a commercially available, chronically sensing DBS device, we show that the amplitude of beta oscillations in the STN of PD patients follows a consistent diurnal pattern even while patients are undergoing continuous DBS. Moreover, we report that it is possible for common movements to confound the on-device beta power measure. Our approach highlights the value of taking a longer-timescale perspective on data from DBS devices and has important implications for the future implementation of aDBS.

In our group of patients, STN beta power showed consistent diurnal fluctuations at an individual patient level during continuous high-frequency DBS. Moreover, across patients, the beta was consistently increased during the day, and reduced during the night. The common pattern of beta modulation closely followed the estimated sleep schedule of patients. Such a contrast between sleeping and waking epochs is consistent with previous comparisons of sleeping and waking beta activity in PD patients^20,22,23^ and confirms that beta power differences between sleeping and waking remain prominent even under a regime of continuous high-frequency DBS. This is in line with electrophysiological recordings of STN LFPs showing that DBS reduces beta power and/or shortens beta bursts, but does not eliminate beta activity completely^7,14^. Our results indicate that beta during sleep is considerably lower than this residual activity.

The reduction in beta during sleep may be explained by changes in the physiological mechanisms underlying the generation of beta oscillations during slow-wave sleep. Whole-night recordings of STN LFPs show that the beta frequency activity is reduced by around half in NREM sleep stages compared to REM and wakefulness^22,23^. In contrast, slow and delta frequencies increase, in line with the key role of these frequencies in thalamocortical sleep structure^38^. In parkinsonian rodents recorded under anesthesia, the emergence of beta oscillations across the cortex, basal ganglia and thalamus is dependent on the absence of slow-wave activity^39–41^. It should be noted, however, that during NREM sleep in parkinsonian primates, beta oscillations are interspersed with slow oscillations and such episodes of increased beta activity may underlie sleep disturbances^42^. In our study, all beta power measurements were conducted during continuous DBS, which has previously been shown to improve sleep quality^43^.

For most patients, there were still significant effects of time of day within estimated sleep or waking epochs. Examples of this can be found in the individual patient beta profiles provided in Supplementary Figs. 1–6. These could be driven by circadian oscillators^26,27^, though some of the more idiosyncratic profiles might be more readily explained by dopaminergic medication regime^3,10^ or individual diurnal behavior patterns.

It is important to emphasize that our effects were observed using measures of beta band power that were collected on-device in a 5 Hz window around the beta peak determined by a clinician. To build up a higher resolution picture of the diurnal evolution of beta activity and its relationship with other neural dynamics over the course of the day, the use of devices with the capacity to export full LFP streams will be required. Characterization of such wide-band signals over multiple nights, together with detailed polysomnography and sleep diaries, are needed to fully understand how beta, NREM activity, and sleep quality are related in PD.

Further clinical studies with continuous monitoring of patient experience and symptoms are needed to establish whether future aDBS implementations would benefit from an awareness of the diurnal rhythmicity in beta we report here. For instance, while beta activity is reduced during sleep, STN DBS is still reported to lead to improved sleep in PD patients^43^. Therefore, it is potentially unhelpful for DBS to be fully disengaged during sleep epochs. To provide an optimal stimulation regime for round-the-clock symptom control, it might be desirable to incorporate online sleep detection^24^ or an internal representation of a patient’s individual “chronotype” across the full diurnal cycle^44^ into the DBS control system.

We have recently highlighted the propensity of electrocardiogram (ECG) artifacts to corrupt signals recorded from devices with chronic sensing capability^34^. Heart rate is modulated by the brain’s internal clock, slowing during the night and gradually rising to a peak in the afternoon^45^, and could thus contribute to the diurnal fluctuations seen here. By using the Perceive toolbox to screen our data, we were able to exclude ECG as a major contributing factor here. In contrast, delineating the influence of movement artifacts on chronic sensing data is more challenging. We showed that LFP streams from the Percept DBS implant can be heavily affected by common movements. Head movements, in particular, yielded band power estimates above resting levels across patients. To address the influence of these artifacts, we interrogated the interdependence of theta and beta amplitude across hemispheres. If broadband movement or ECG artifacts were the primary drivers of diurnal modulation in both hemispheres, theta and beta should have been highly correlated and theta would be similarly or more strongly locked to time of day. However, in most patients, the correlation between bands was either weakly positive or negative, and beta power, on average, showed a stronger influence on the time of day. Moreover, our characterization of beta amplitude during movement was made over short LFP segments (the seconds surrounding the movement of interest) while long-term sensing with the Percept generates a measure of average power over much longer epochs (10 min). Therefore, the impact of short, isolated movements on the long-term sensing data may be modest in most patients. Nevertheless, the streamed signal is vulnerable to influence from such movements if they are repeated or sustained during a large proportion of a 10-min epoch. This is highlighted in our recordings from a patient with regular episodes of dyskinesia (see Fig. 5). Overall, it is likely that, in most patients, diurnal fluctuations in the streamed signal reflect physiological changes in LFP beta power. However, if artifacts associated with specific movements occur frequently enough and cluster together, they may significantly confound the signal.

**Fig. 6:**
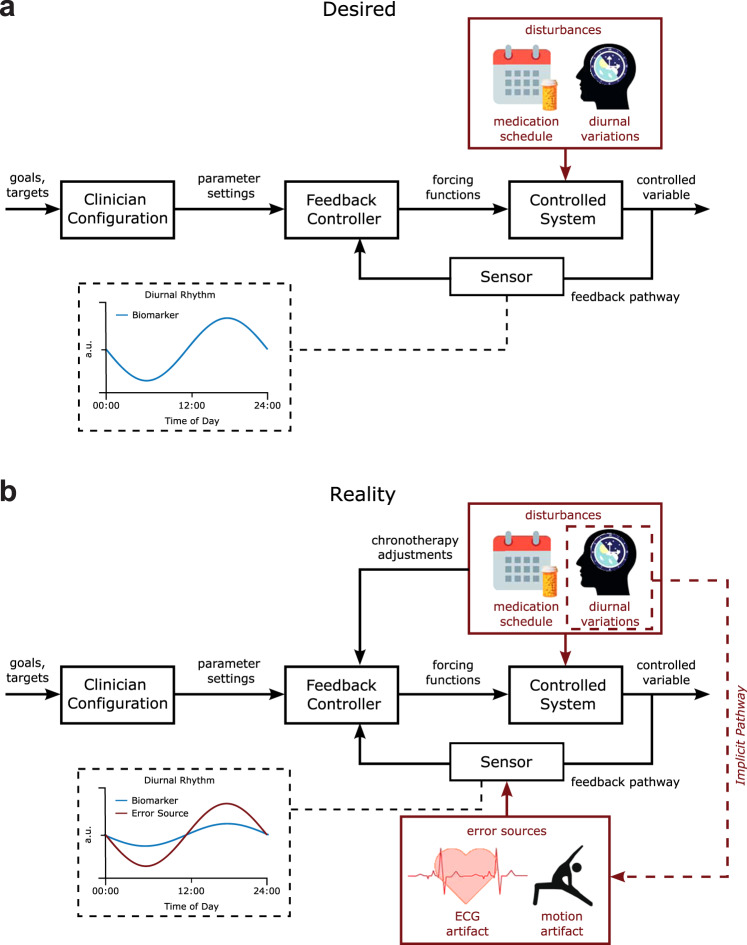
Desired vs. realistic block diagram of chronoadaptive feedback DBS control system. **a** Desired control system. In the desired DBS control system, therapeutic goals (i.e., % suppression of beta power) are specified by clinicians for specific times of the day. A feedback controller modulates the DBS parameters to maintain the controlled variable, the biomarker, at the specified target level. Time-based adjustments of control goals synchronized to patient-specific chronotypes can then be incorporated to accommodate known disturbances such as the medication-schedule and diurnal variation of the monitored biomarker. In this manner, time-localized adjustments to stimulation parameters are provided in response to physiological variations. **b** Actual control system. In the actual control system at present diurnal variations in artifact sources may couple into the sensor in the feedback pathway. This can confound observations of physiological fluctuations in the monitored biomarker signal due to the amplitude of artifacts being greater than the signal of interest.

While in an idealized scenario the input to an aDBS control loop is driven by physiological changes in pathological beta amplitude (Fig. 6a), our results highlight that as patients go about their daily lives, they will behave in ways that have the potential to drive non-physiological fluctuations in the streamed amplitude data. Low-frequency oscillations, including the beta range, appear particularly vulnerable to ECG and movement artifacts. In terms of threshold-based adaptive stimulation, this could lead to a situation whereby the threshold for stimulation is set in the “desired” condition but is then inappropriately triggered in real-life conditions when artifacts are present (Fig. 6b). In PD, movement artifacts that constantly push the signal above the trigger threshold would result in continuous stimulation. This would mimic conventional continuous DBS, and any potential benefits of adaptive stimulation (e.g., side effect control) would be lost, a situation that appears particularly likely to occur in patients with dyskinesia. Movement-based confounds could be more serious in applications where dual-threshold aDBS is implemented with a high upper stimulation amplitude, where movement artifacts could maintain a sub-optimally high level of stimulation. To further complicate these issues, physiological circadian or diurnal rhythms are implicitly synchronized to the timescale of ECG and movement artifacts through physical activity. The contribution of these artifacts will differ based on the signal-to-noise ratio of the movement artifact and the physiological signal. For example, in some patients, beta amplitude in sleep may be physiologically driven, but in wakefulness be a composite of physiology and artifact (Fig. 6b).

As the use of devices with sensing capability develops, it will be crucial to evaluate the composite of physiological and artifactual influences on any input signal used either to investigate pathophysiological and/or to control stimulation. Pre-screening for artifact contamination from the ECG or movements is an important first step toward this. Ideally, access to the full-spectrum LFP signal underlying the continuous band power measurements would provide clinicians and researchers with a means to evaluate the integrity of the data. When using devices without this functionality, regular inspection of the distribution of values returned by the system may provide an indication of contamination, as in the dyskinetic patient described here. When the sensing is used for aDBS, systems should be set up in such a way that transient or prolonged artifact influence cannot raise or lower overall stimulation amplitude to dangerous or poorly tolerated levels^46^. Finally, online detection of extreme values or a suspicious measurement distribution could be set to engage a pre-determined fail-safe mode, reverting the system to a well-tolerated level of stable DBS. Development of cranially mounted devices may further help to avoid ECG and movement artifacts^34,35^.

In conclusion, beta activity in PD shows significant diurnal fluctuations, with a reduction at night that may decrease aDBS to suboptimal stimulation levels. Moreover, artifacts resulting from patient activities remain a concern for applications of chronic sensing. Nevertheless, chronic recordings of band-specific LFP amplitude provide the opportunity to define the chronotype of a disease-relevant activity. Access to this information provides many opportunities to improve therapy, both through better-informed adjustments made by the clinician and through the development of feedforward and feedback strategies to improve adaptive DBS.

## Methods

### Participants

Written informed consent was obtained from all patients included in this study. The study was approved by the ethical review board of Charité Universitätsmedizin Berlin (EA2/256/60) and adhered to the tenets of the Declaration of Helsinki.

Eleven patients with advanced PD implanted with bilateral electrodes for STN DBS and a Percept IPG (Medtronic, Minneapolis, MN, USA) capable of chronic sensing participated in the study (surgery as previously described in ref. ^47^). Patients 1–7 were implanted with model 3389 leads (Medtronic), and patients 8–11 were implanted with Medtronic SenSight electrodes (model B33005). Patients 1–6 (age 61.2 ± 4.7 years; disease duration 10.8 ± 3.7 years; three females) were included in the long-term bilateral beta monitoring data set, while patients 7–11 (age 65.0 ± 8.3; disease duration 13.6 ± 3.0 years; one female) were included in the simultaneous contralateral beta and theta power monitoring data set. Clinical severity and therapeutic effect were assessed using the MDS-Unified Parkinson’s Disease Rating Scale (UPDRS)-Part III. Prior to DBS implantation, motor impairment significantly improved with medication with an average UPDRS-III change of 50 ± 18%. Postoperatively, all patients benefited from chronic STN DBS with a mean improvement in UPDRS ON DBS/OFF medication of 43 ± 16% (*n* = 7, patients 8–11 have not yet passed the follow-up). Full clinical details are provided in Table 2.

**Table 2:** Clinical information and recording details.

ID	Age range (y)	Sex	Disease duration (y)	UPDRS pre-DBS OFF/ON MED	UPDRS OFF/ON DBS, OFF MED at 3 months follow-up	Time of recording (days post-surgery [duration])	Stimulation amplitude (mA)	Peak frequency (Hz)	LEDD at time of recording
1	61–65	F	10	50/9	22/15	43–74 (31)	R:2.2–2.8 L:0.1–0.2	R: 25.39 L: 19.53	575 mg
2	66–70	M	11	63/30	48/34	123–155 (32)	R:0.5 L:0.5	R: 27.34^c^ L: 16.60	1175 mg
3	56–60	F	15	37/23	29/20	32–64 (32)	R:1.4–1.6 L:1.4–1.8	R:25.39 L:13.67	100 mg
4	61–65	F	6	50/33	40/19	73–91 (18)	R: 1 L: 1	R: 14.65 L: 18.55^c^	300 mg
5	66–70	M	15	66/27	39/14	37–96 (59) +21^b^	R:2–2.8	R: 15.63	300 mg
6	56–60	M	8	23/4	28/11	116–148 (32)	R:1.7–2 L:1.7–2	R:22.46 L: 20.51	175 mg
7	56–60	M	11	72/39	47/33^a^	393–396 (3)	R: 1.2 L: 2	R: 20.51 L: 6.84	711 mg
8	66–70	F	11	49/30	N/A	10–20 (10)	R:0-1 L:0-1	R: 23.44 L: 7.81	580 mg
9	71–75	M	15	67/43	N/A	8–19 (11)	R: 0.5–1.5 L: 0.5–1.5	R: 15.63 L: 7.81	575 mg
10	66–70	M	18	40/25	N/A	8–14 (6)	R:0.5 L:0.5	R: 7.81 L: 13.67	800 mg
11	56–60	M	13	68/39	N/A	10–14 (4)	R: 1 L: 1	R: 7.81 L: 13.67	900 mg

Information on the participants included in this study. Age at surgery, sex (F female, M male), disease duration in years. MDS-UPDRS Part III for OFF medication/ON medication state at the pre-DBS evaluation and during OFF Medication/DBS OFF/ON at 3 months follow-up for patients 1–6 and 12 months follow-up for patient 7. Patients 1–6 were included in the long-term analysis, while in patients 7–11 beta/theta band activity was recorded simultaneously around the 12 months follow-up (patient 7) or in a subacute post-surgical phase (patients 8–11). Accordingly, LEDD are presented at the time of discharge from the 12 months follow-up for patient 7, after surgery for patients 8–11 and at the admission for 3 months follow-up for patients 1–6. Stimulation amplitude was either set to a single value or was allowed to vary within the given range during the recording period. Peak frequency for chronic sensing was set within the beta range on all STNs under investigation for patients 1–6, while for patients 7–11 one STN was recorded in the theta frequency range respectively.

^a^At 12 months follow-up.

^b^Additional data were collected to investigate beta power across sleeping and waking periods (not in the main data set).

^c^STN was excluded from analysis because of ECG contamination.

### Sensing

Frequencies for chronic recording were visually selected in power spectra generated by the BrainSense signal test, which automatically computes a fast Fourier transform of 20-s LFP recordings from the possible bipolar recording configurations per hemisphere. The recording contacts with the most prominent beta peak were selected. A window of 5 Hz around this peak was used for long-term sensing of peak power (µVp) as an average every 10 min, with associated time stamps in coordinated universal time (UTC). As data were collected in Germany in two different time zones (Central European Time and Central European Summer Time), time stamps were corrected to reflect local time. In patients 1–6, beta power was collected for an average of 34 ± 13.4 days at a stable optimized medication and stimulation regime until the 3-month follow-up. Patients 7–11 were included for the data set exploring frequency-specificity of the beta band fluctuations in subacute recordings after DBS surgery. Peak beta frequency was selected as described above in one STN, while in the contralateral STN peak power was logged in a 5 Hz window around a theta frequency (7.61 ± 0.43 Hz) for 6.8 ± 3.6 days, irrespective of whether an oscillatory peak was present. Medication and stimulation amplitude were adjusted as per the standard of care. Stimulation amplitudes and sensing frequencies for all patients are provided in Table 2. Stimulation amplitude over time for patients 7–11 is provided in Supplementary Fig. 7.

### Assessing the impact of movements and medication on the LFP signal

Additional recordings during different types of movement were performed in patients 3, 4, 5, and 6 while they were on their usual medication. LFPs were recorded using the Percept IPG, sampled at 250 Hz during DBS, streamed to the Medtronic clinician programmer, and exported to the json-file format. After a rest recording of 3 min in a sitting position, patients were asked to perform head movements (nodding (24 ± 10.98 s), shaking (24.63 ± 8.81 s), tilting to the sides (27.63 ± 10.31 s)), arm movements (swinging back and forth (29.86 ± 2.84 s), lifting the arms to the sides (30.36 ± 3.5 s), closing the hands in front of the chest (36.25 ± 18.37 s)), and axial movements (chest rotation (29.13 ± 2.84 s), bending backward and forwards (31 ± 10.12 s)). Patients also changed position (sitting (15.92 ± 6.00 s), standing up (2.25 ± 0.40 sec), standing (15.79 ± 6.57 s), and sitting down (2.71 ± 0.75 s), at least three times) and walked back and forth (walking (26.13 ± 4.13 s) and turning (2.63 ± 0.25 s)). Video recordings of the patients were registered to the LFP signal by videotaping the start of each LFP recording on the programmer tablet. Subsequently, time stamps for each movement were aligned to the LFP signal. Any ECG artifacts were detected and removed from the LFP streams using the Perceive toolbox^34^. Signal power estimates were calculated after the application of a low-pass filter at 98 Hz and a high-pass filter at 4 Hz for each movement epoch type, with the beta band set as a 5 Hz window around the STN-specific beta peak used for long-term sensing, while theta band estimates were calculated as band power between 4–8 Hz. For the comparison of in-clinic daytime beta power with the out-of-clinic long-term measurements, additional 60-s at-rest LFP recordings were obtained from patients 1 and 3 after at least 12 h withdrawal of dopaminergic medication and after controlled administration of fast-acting levodopa (100–200 mg l-dopa 30+ min prior to recording), on and off DBS.

### Data analysis

Analysis was carried out using custom-written scripts in MATLAB (Mathworks, Natick, MA, USA) and with the “Circa Diem” analysis toolbox developed by author JJvR (v0.1, available on https://github.com/joramvanrheede/circa_diem, 10.5281/zenodo.5961105). ECG artifact influences were examined using the open-source Perceive Toolbox (https://github.com/neuromodulation/perceive/^34^). Means are reported as mean ± standard deviation and compared using paired two-tailed *t*-tests if normally distributed, or using two-tailed Wilcoxon signed-rank tests if not (relevant data were tested for normality using Lilliefors’ goodness-of-fit test).

### Detection of ECG artifact contamination and cleaning of LFP streams

The LFP streams from the BrainSense signal test for beta peak detection were screened for contamination by cardiac signals using the Perceive toolbox^34^. If an ECG artifact was identified by the classifier or observed on visual inspection, the STN long-term data were excluded, resulting in the removal of two STN time series (patient 2 right STN; patient 4 left STN). The ECG artifact analysis results for all six long-term bilateral sensing patients are included in Supplementary Figs. 1–6a–c, h–j.

### Outlier removal, data normalization, and de-trending

The LFP band power measure contained occasional extreme values. As the Percept device was only able to log a band power summary measure and not the raw data, inspection or recovery of the underlying LFP was not possible. We, therefore, removed outliers by replacing any values with a *z*-score greater than 6 with a value obtained from linear interpolation between their nearest neighbor values. This process was repeated until the data set contained no values with a *z*-score greater than 6. In the long-term beta monitoring data set, the mean number of outliers per data set was 18 ± 23, and the maximum number of outliers for a time series was 71 (~0.82% of data points over 59 days). For visual comparison between patients and hemispheres, long-term sensing data are sometimes presented as *z*-scores. For visualization of diurnal patterns in heatmaps and rose plots, and to investigate diurnal correlations between beta and theta power while removing any influence of longer-term drift from the correlation measure, time series are detrended with the Circa Diem toolbox where indicated. De-trending was accomplished by dividing all LFP power measurements for a given day by that day’s median value (such that in the detrended data, the median for each day is 1).

### Time of day fits, variance explained by time of day, and temporal shuffling test

To estimate the effect of time of day on the variance of the LFP power time series, we created a fit based on the average values for each time of day. The day was divided into 30-minute segments, and the mean for this time bin was used as the fit estimate. The fit estimate for any value between the centers of time bins was then the result of linear interpolation. The influence of time of day on the signal could now be “removed” by subtracting the fit value for each time of day from the observed values. The variance explained by time of day was then calculated by taking the total variance of the time series, subtracting the variance remaining after the influence of time of day was removed, and then dividing by the total variance. To estimate how likely it was that a given value might be observed by chance, we devised a temporal shuffling test: we generated 1000 shuffled data sets by applying random shifts between 0 and 24 h to the data for each day in a data set such that days were offset relative to each other but the time series for each day maintained its temporal structure (values that exceeded 24 h were moved to the beginning of the day). This resulted in a null distribution of values for variance explained by time of day, to which the values obtained from the real data set could be compared. Time of day fits, the calculation of variance explained by time of day, and the temporal shuffling test are all implemented in the Circa Diem toolbox.

### Estimates of sleeping and waking times and aligning data to estimated rise time

To examine beta power changes across periods of sleeping and waking, we acquired 21 days of additional bilateral STN data from patient 5, who was asked to log their bedtime and rise time each day. We approximated wake-up time in other patients by aligning their data to their first scheduled medication intake. To estimate the effect of time of day during sleeping or waking hours only, we separated estimated sleeping and waking epochs for all patients during the long-term data acquisition by selecting a time window usually dominated by sleep (00:00–06:00) and a time window usually dominated by waking (08:00–20:00). We used the same time windows to obtain distributions of long-term STN LFP power values during estimated day or night-time, for comparison with the daytime in-clinic measurements on and off medication and DBS.

## Supplementary information

Supplementary Figures

## Data Availability

The primary data set for this publication is held by the Movement Disorder and Neuromodulation Unit, Department of Neurology, Charité Univesitätsmedizin Berlin, Germany, and access requests can be addressed to Prof. Dr. Andrea A. Kühn, andrea.kuehn@charite.de. Code and derivative data produced for this study are held by the MRC Brain Network Dynamics Unit at the University of Oxford https://www.mrcbndu.ox.ac.uk/data-sharing-policy and have not been made available at this time because they are being used for ongoing projects, but requests can be addressed to the Data Access Committee via andrew.sharott@bndu.ox.ac.uk.

## Code availability

The Circa Diem MATLAB toolbox for the analysis of diurnal properties of time series and/or event data generated for this study by author J.J.v.R. is available on https://github.com/joramvanrheede/circa_diem and 10.5281/zenodo.5961105. Custom data analysis and visualization scripts will be made available by the authors upon reasonable request.

## References

[1] Deuschl G (2006). A randomized trial of deep-brain stimulation for Parkinson’s disease. N. Engl. J. Med..

[2] Schuepbach WMM (2013). Neurostimulation for Parkinson’s disease with early motor complications. N. Engl. J. Med..

[3] Brown P (2001). Dopamine dependency of oscillations between subthalamic nucleus and pallidum in Parkinson’s disease. J. Neurosci..

[4] Krauss JK (2021). Technology of deep brain stimulation: current status and future directions. Nat. Rev. Neurol..

[5] Kühn AA, Volkmann J (2017). Innovations in deep brain stimulation methodology. Mov. Disord..

[6] Hammond C, Bergman H, Brown P (2007). Pathological synchronization in Parkinson’s disease: networks, models and treatments. Trends Neurosci..

[7] Kühn AA (2008). High-frequency stimulation of the subthalamic nucleus suppresses oscillatory activity in patients with Parkinson’s disease in parallel with improvement in motor performance. J. Neurosci..

[8] Kühn AA, Kupsch A, Schneider G, Brown P (2006). Reduction in subthalamic 8–35Hz oscillatory activity correlates with clinical improvement in Parkinson’s disease. Eur. J. Neurosci..

[9] Feldmann LK (2022). Toward therapeutic electrophysiology: beta-band suppression as a biomarker in chronic local field potential recordings. Npj Park Dis..

[10] Tinkhauser G (2017). Beta burst dynamics in Parkinson’s disease OFF and ON dopaminergic medication. Brain J. Neurol..

[11] Arlotti M (2018). Eight-hours adaptive deep brain stimulation in patients with Parkinson disease. Neurology.

[12] Bocci T (2021). Eight-hours conventional versus adaptive deep brain stimulation of the subthalamic nucleus in Parkinson’s disease. Npj Park Dis..

[13] Little S (2013). Adaptive deep brain stimulation in advanced Parkinson disease. Ann. Neurol..

[14] Tinkhauser G (2017). The modulatory effect of adaptive deep brain stimulation on beta bursts in Parkinson’s disease. Brain.

[15] Swann NC (2018). Adaptive deep brain stimulation for Parkinson’s disease using motor cortex sensing. J. Neural. Eng..

[16] Feldmann LK (2021). Subthalamic beta band suppression reflects effective neuromodulation in chronic recordings. Eur. J. Neurol..

[17] Neumann W-J (2017). Long term correlation of subthalamic beta band activity with motor impairment in patients with Parkinson’s disease. Clin. Neurophysiol..

[18] Velisar A (2019). Dual threshold neural closed loop deep brain stimulation in Parkinson disease patients. Brain Stimul..

[19] Cummins DD (2021). Chronic sensing of subthalamic local field potentials: comparison of first and second generation implantable bidirectional systems within a single subject. Front. Neurosci..

[20] Gilron R (2021). Long-term wireless streaming of neural recordings for circuit discovery and adaptive stimulation in individuals with Parkinson’s disease. Nat. Biotechnol..

[21] Zahed H (2021). The neurophysiology of sleep in Parkinson’s disease. Mov. Disord..

[22] Thompson JA (2018). Sleep patterns in Parkinson’s disease: direct recordings from the subthalamic nucleus. J. Neurol. Neurosurg. Psychiatry.

[23] Urrestarazu E (2009). Beta activity in the subthalamic nucleus during sleep in patients with Parkinson’s disease. Mov. Disord..

[24] Gilron R (2021). Sleep-aware adaptive deep brain stimulation control: Chronic use at home with dual independent linear discriminate detectors. Front. Neurosci..

[25] Kim P (2019). Coupling the circadian clock to homeostasis: the role of period in timing physiology. Endocr. Rev..

[26] Hastings MH, Maywood ES, Brancaccio M (2018). Generation of circadian rhythms in the suprachiasmatic nucleus. Nat. Rev. Neurosci..

[27] Paul JR (2020). Circadian regulation of membrane physiology in neural oscillators throughout the brain. Eur. J. Neurosci..

[28] Gaggioni G, Maquet P, Schmidt C, Dijk D-J, Vandewalle G (2014). Neuroimaging, cognition, light and circadian rhythms. Front. Syst. Neurosci..

[29] Kühn AA (2004). Event‐related beta desynchronization in human subthalamic nucleus correlates with motor performance. Brain.

[30] Lofredi R (2019). Beta bursts during continuous movements accompany the velocity decrement in Parkinson’s disease patients. Neurobiol. Dis..

[31] Neuville RS (2021). Differential effects of pathological beta burst dynamics between Parkinson’s disease phenotypes across different movements. Front. Neurosci..

[32] Jenkinson N, Brown P (2011). New insights into the relationship between dopamine, beta oscillations and motor function. Trends Neurosci..

[33] Quinn EJ (2015). Beta oscillations in freely moving Parkinson’s subjects are attenuated during deep brain stimulation. Mov. Disord..

[34] Neumann W-J (2021). The sensitivity of ECG contamination to surgical implantation site in brain computer interfaces. Brain Stimul..

[35] Sorkhabi MM, Benjaber M, Brown P, Denison T (2020). Physiological artifacts and the implications for brain-machine-interface design. IEEE Int. Conf. Syst. Man Cybern..

[36] Swann NC (2018). Chronic multisite brain recordings from a totally implantable bidirectional neural interface: experience in 5 patients with Parkinson’s disease. J. Neurosurg..

[37] Hammer LH, Kochanski RB, Starr PA, Little S (2022). Artifact characterization and a multipurpose template-based offline removal solution for a sensing-enabled deep brain stimulation device. Stereotact Funct Neurosurg..

[38] Steriade M, McCormick DA, Sejnowski TJ (1993). Thalamocortical oscillations in the sleeping and aroused brain. Science.

[39] Mallet N (2008). Disrupted dopamine transmission and the emergence of exaggerated beta oscillations in subthalamic nucleus and cerebral cortex. J. Neurosci..

[40] Sharott A, Vinciati F, Nakamura KC, Magill PJ (2017). A population of indirect pathway striatal projection neurons is selectively entrained to parkinsonian beta oscillations. J. Neurosci..

[41] Nakamura KC, Sharott A, Tanaka T, Magill PJ (2021). Input zone-selective dysrhythmia in motor thalamus after dopamine depletion. J. Neurosci..

[42] Mizrahi-Kliger AD, Kaplan A, Israel Z, Deffains M, Bergman H (2020). Basal ganglia beta oscillations during sleep underlie Parkinsonian insomnia. Proc. Natl Acad. Sci. USA.

[43] Baumann-Vogel, H. et al. The impact of subthalamic deep brain stimulation on sleep–wake behavior: A prospective electrophysiological study in 50 Parkinson patients. *Sleep* **40**, (2017). 10.1093/sleep/zsx033 28369624

[44] Fleming JE (2022). Embedding digital chronotherapy into bioelectronic medicines. Iscience.

[45] Black N (2019). Circadian rhythm of cardiac electrophysiology, arrhythmogenesis, and the underlying mechanisms. Heart Rhythm.

[46] Gunduz A (2019). Adding wisdom to ‘smart’ bioelectronic systems: a design framework for physiologic control including practical examples. Bioelectron. Med..

[47] Feldmann LK, Neumann W-J, Faust K, Schneider G-H, Kühn AA (2021). Risk of infection after deep brainstimulation surgery with externalization and local-field potential recordings:Twelve-year experience from a single institution. Stereot. Funct. Neurosurg.

