# Supplementary material for: Diurnal modulation of subthalamic beta oscillatory power in Parkinson’s disease patients during deep brain stimulation
Source: NPJ Parkinsons Dis. 2022 Jul 8;8:88. doi: 10.1038/s41531-022-00350-7 (PMC9270436; doi:10.1038/s41531-022-00350-7)
Supplement: Supplementary file 1 — Supplementary Figures [file 41531_2022_350_MOESM1_ESM.pdf]

Supplementary Figure 1: Patient #1 individual data overview

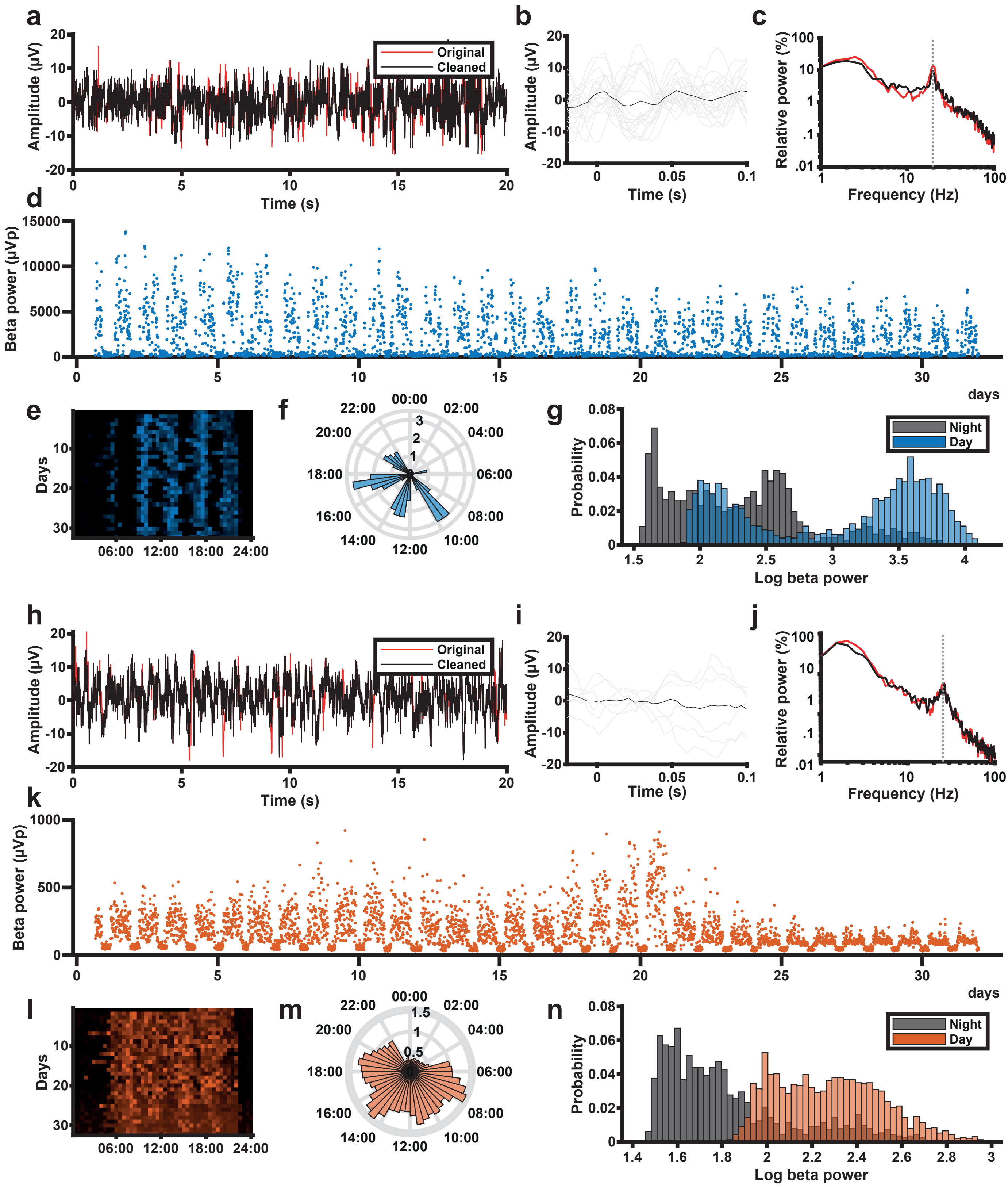

Supplementary Figure 1: Patient #1 individual data overview

a,h: LFP recorded from the left (**a**) and right (**h**) STN of a PD patient during the Percept's BrainSense signal test. Red line represents the original signal, black represents the signal that has been recovered using the Perceive toolbox (34). **b,i:** ECG waveforms detected in the LFP signals in **a**&**h** using the Perceive toolbox. Grey lines represent individual candidate wave-forms, the black line represents the average waveform estimate. No consistent ECG waveform was found in either **a** or **h**. **c,j:** Normalised Welch's power spectra of the original (red) and cleaned (black) LFP signals, with dotted line indicating the clinician-set beta center frequency. **d,k:** Beta power values (µVp) recorded from the left (**d**) and right (**k**) STN of this PD patient during the long-term data collection period (outliers with z-score > 6 removed). **e,l:** Heat map of beta power (detrended by normalising each day to its median value) across the 24 hours of the day for all days in the data collection period, for the same example STN. **f,m:** Detrended beta power across the 24-hour diurnal cycle. For each day, the median beta power was calculated for each 30-minute time bin, and bar height in the circular bar graph represents the median across days. **g,n:** Distributions of daytime (08:00-20:00) and night-time (00:00-06:00) beta power measurements, log-transformed (log10(µVp)).

Supplementary Figure 2: Patient #2 individual data overview

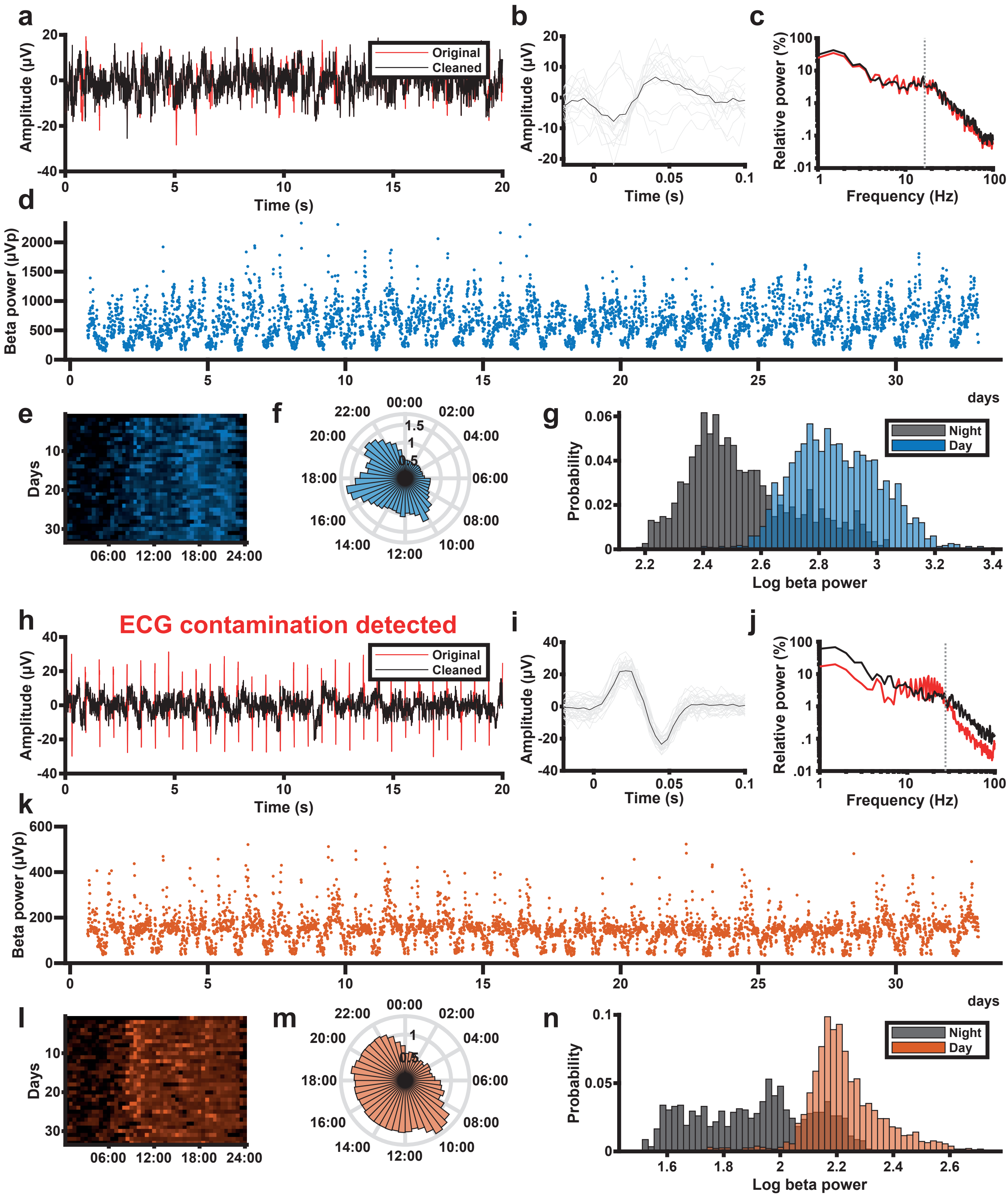

Supplementary Figure 2: Patient #2 individual data overview

a,h: LFP recorded from the left (**a**) and right (**h**) STN of a PD patient during the Percept's BrainSense signal test. Red line represents the original signal, black represents the signal that has been recovered using the Perceive toolbox (34). **b,i:** ECG waveforms detected in the LFP signals in **a**&**h** using the Perceive toolbox. Grey lines represent individual candidate waveforms, the black line represents the average waveform estimate. No consistent ECG waveform was found in **a**, but the LFP in **h** showed ECG contamination and this hemisphere was excluded from further analysis. **c,j:** Normalised Welch's power spectra of the original (red) and cleaned (black) LFP signals, with dotted line indicating the clinician-set beta center frequency. **d,k:** Beta power values (μVp) recorded from the left (**d**) and right (**k**) STN of this PD patient during the long-term data collection period (outliers with z-score > 6 removed). **e,l:** Heat map of beta power (detrended by normalising each day to its median value) across the 24 hours of the day for all days in the data collection period, for the same example STN. **f,m:** Detrended beta power across the 24-hour diurnal cycle. For each day, the median beta power was calculated for each 30-minute time bin, and bar height in the circular bar graph represents the median across days. **g,n:** Distributions of daytime (08:00-20:00) and night-time (00:00-06:00) beta power measurements, log-transformed (log10(μVp)).

Supplementary Figure 3: Patient #3 individual data overview

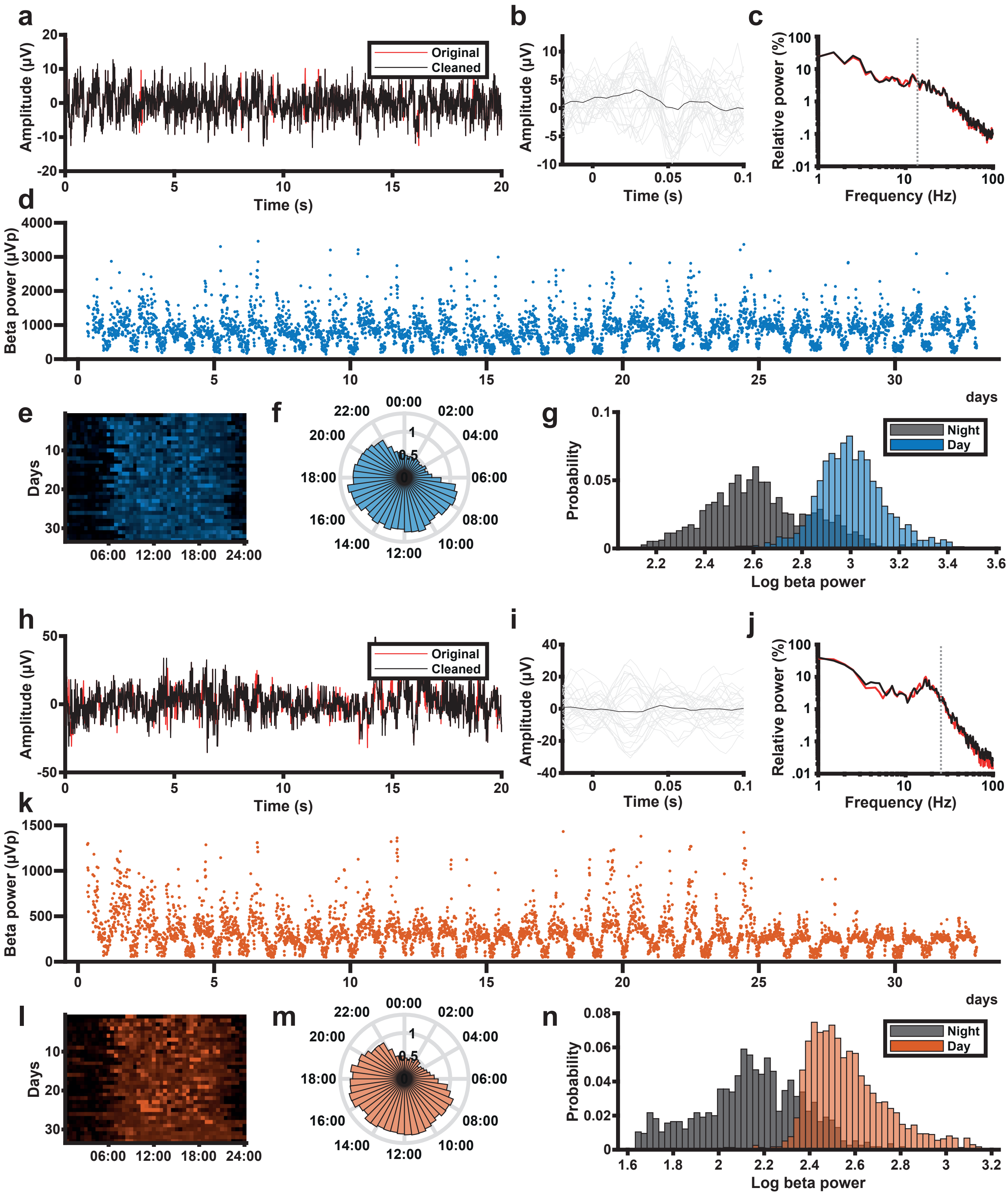

Supplementary Figure 3: Patient #3 individual data overview

a,h: LFP recorded from the left (**a**) and right (**h**) STN of a PD patient during the Percept's BrainSense signal test. Red line represents the original signal, black represents the signal that has been recovered using the Perceive toolbox (34). **b,i:** ECG waveforms detected in the LFP signals in **a**&**h** using the Perceive toolbox. Grey lines represent individual candidate waveforms, the black line represents the average waveform estimate. No consistent ECG waveform was found in either **a** or **h**. **c,j:** Normalised Welch's power spectra of the original (red) and cleaned (black) LFP signals, with dotted line indicating the clinician-set beta center frequency. **d,k:** Beta power values (µVp) recorded from the left (**d**) and right (**k**) STN of this PD patient during the long-term data collection period (outliers with z-score > 6 removed). **e,l:** Heat map of beta power (detrended by normalising each day to its median value) across the 24 hours of the day for all days in the data collection period, for the same example STN. **f,m:** Detrended beta power across the 24-hour diurnal cycle. For each day, the median beta power was calculated for each 30-minute time bin, and bar height in the circular bar graph represents the median across days. **g,n:** Distributions of daytime (08:00-20:00) and night-time (00:00-06:00) beta power measurements, log-transformed (log10(µVp)).

Supplementary Figure 4: Patient #4 individual data overview

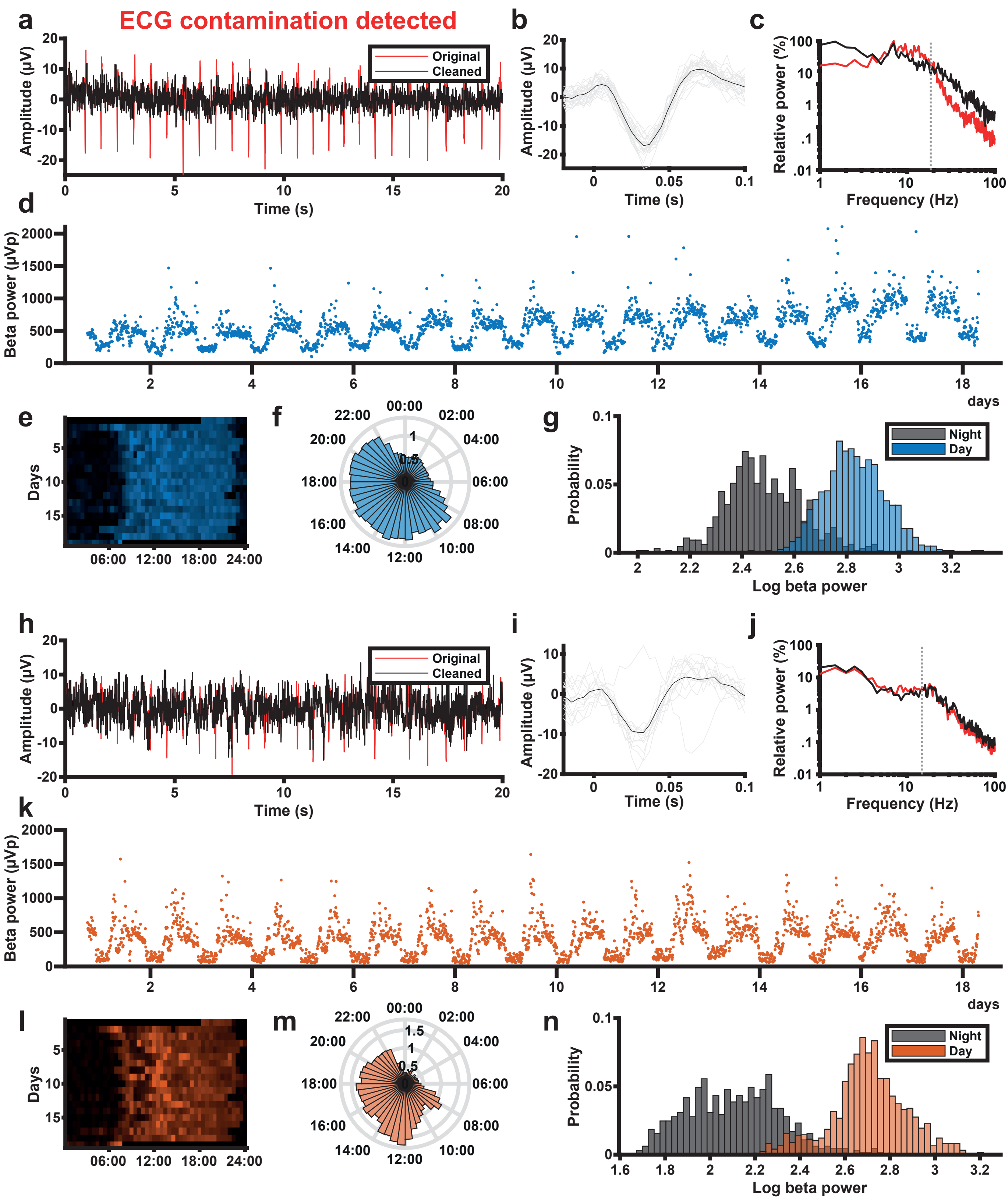

Supplementary Figure 4: Patient #4 individual data overview
a,h: LFP recorded from the left (**a**) and right (**h**) STN of a PD patient during the Percept's BrainSense signal test. Red line represents the original signal, black represents the signal that has been recovered using the Perceive toolbox (34). **b,i:** ECG waveforms detected in the LFP signals in **a**&**h** using the Perceive toolbox. Grey lines represent individual candidate waveforms, the black line represents the average waveform estimate. The LFP in **a** showed ECG contamination and this hemisphere was excluded from further analysis; no consistent ECG was found in **h**. **c,j:** Normalised Welch's power spectra of the original (red) and cleaned (black) LFP signals, with dotted line indicating the clinician-set beta center frequency. **d,k:** Beta power values (µVp) recorded from the left (**d**) and right (**k**) STN of this PD patient during the long-term data collection period (outliers with z-score > 6 removed). **e,l:** Heat map of beta power (detrended by normalising each day to its median value) across the 24 hours of the day for all days in the data collection period, for the same example STN. **f,m:** Detrended beta power across the 24-hour diurnal cycle. For each day, the median beta power was calculated for each 30-minute time bin, and bar height in the circular bar graph represents the median across days. **g,n:** Distributions of daytime (08:00-20:00) and night-time (00:00-06:00) beta power measurements, log-transformed (log10(µVp)).

Supplementary Figure 5: Patient #5 individual data overview

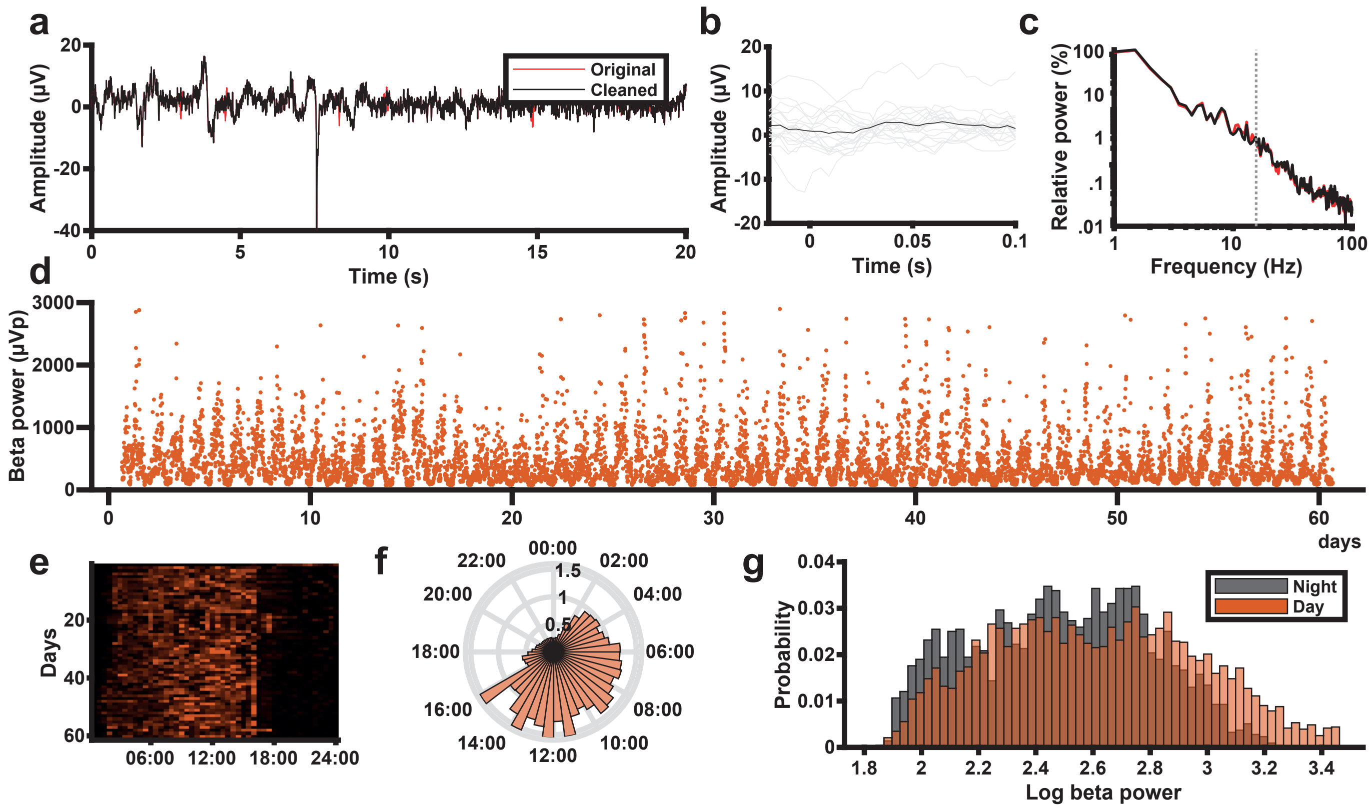

Supplementary Figure 5: Patient #5 individual data overview

a: LFP recorded from the right STN of a PD patient during the Percept's BrainSense signal test. Red line represents the original signal, black represents the signal that has been recovered using the Perceive toolbox (34). **b:** ECG waveforms detected in the LFP signals in **a** using the Perceive toolbox. Grey lines represent individual candidate waveforms, the black line represents the average waveform estimate. No consistent ECG waveform was found in **a**. **c:** Normalised Welch's power spectra of the original (red) and cleaned (black) LFP signals, with dotted line indicating the clinician-set beta center frequency. **d:** Beta power values (µVp) recorded from the right STN of this PD patient during the long-term data collection period (outliers with z-score > 6 removed). **e:** Heat map of beta power (detrended by normalising each day to its median value) across the 24 hours of the day for all days in the data collection period, for the same example STN. **f:** Detrended beta power across the 24-hour diurnal cycle. For each day, the median beta power was calculated for each 30-minute time bin, and bar height in the circular bar graph represents the median across days. **g:** Distributions of daytime (08:00-20:00) and night-time (00:00-06:00) beta power measurements, log-transformed (log10(µVp)).

Supplementary Figure 6: Patient #6 individual data overview

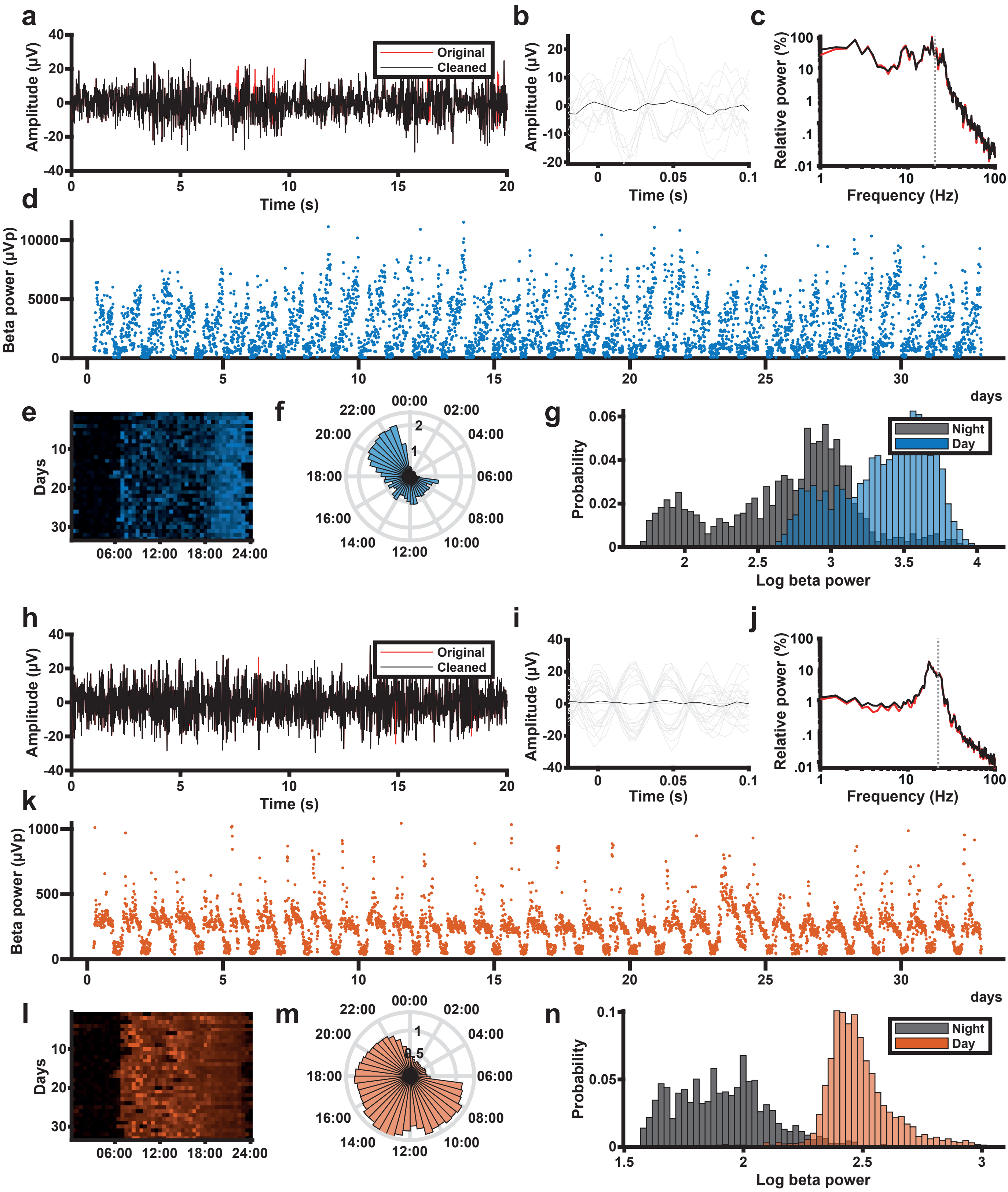

Supplementary Figure 6: Patient #6 individual data overview

a,h: LFP recorded from the left (**a**) and right (**h**) STN of a PD patient during the Percept's BrainSense signal test. Red line represents the original signal, black represents the signal that has been recovered using the Perceive toolbox (34). **b,i:** ECG waveforms detected in the LFP signals in **a**&**h** using the Perceive toolbox. Grey lines represent individual candidate waveforms, the black line represents the average waveform estimate. No consistent ECG waveform was found in either **a** or **h**. **c,j:** Normalised Welch's power spectra of the original (red) and cleaned (black) LFP signals, with dotted line indicating the clinician-set beta center frequency. **d,k:** Beta power values (µVp) recorded from the left (**d**) and right (**k**) STN of this PD patient during the long-term data collection period (outliers with z-score > 6 removed). **e,l:** Heat map of beta power (detrended by normalising each day to its median value) across the 24 hours of the day for all days in the data collection period, for the same example STN. **f,m:** Detrended beta power across the 24-hour diurnal cycle. For each day, the median beta power was calculated for each 30-minute time bin, and bar height in the circular bar graph represents the median across days. **g,n:** Distributions of daytime (08:00-20:00) and night-time (00:00-06:00) beta power measurements, log-transformed (log10(µVp)).

Supplementary Figure 7: Individual beta/theta and stimulation time series for patients 7-11

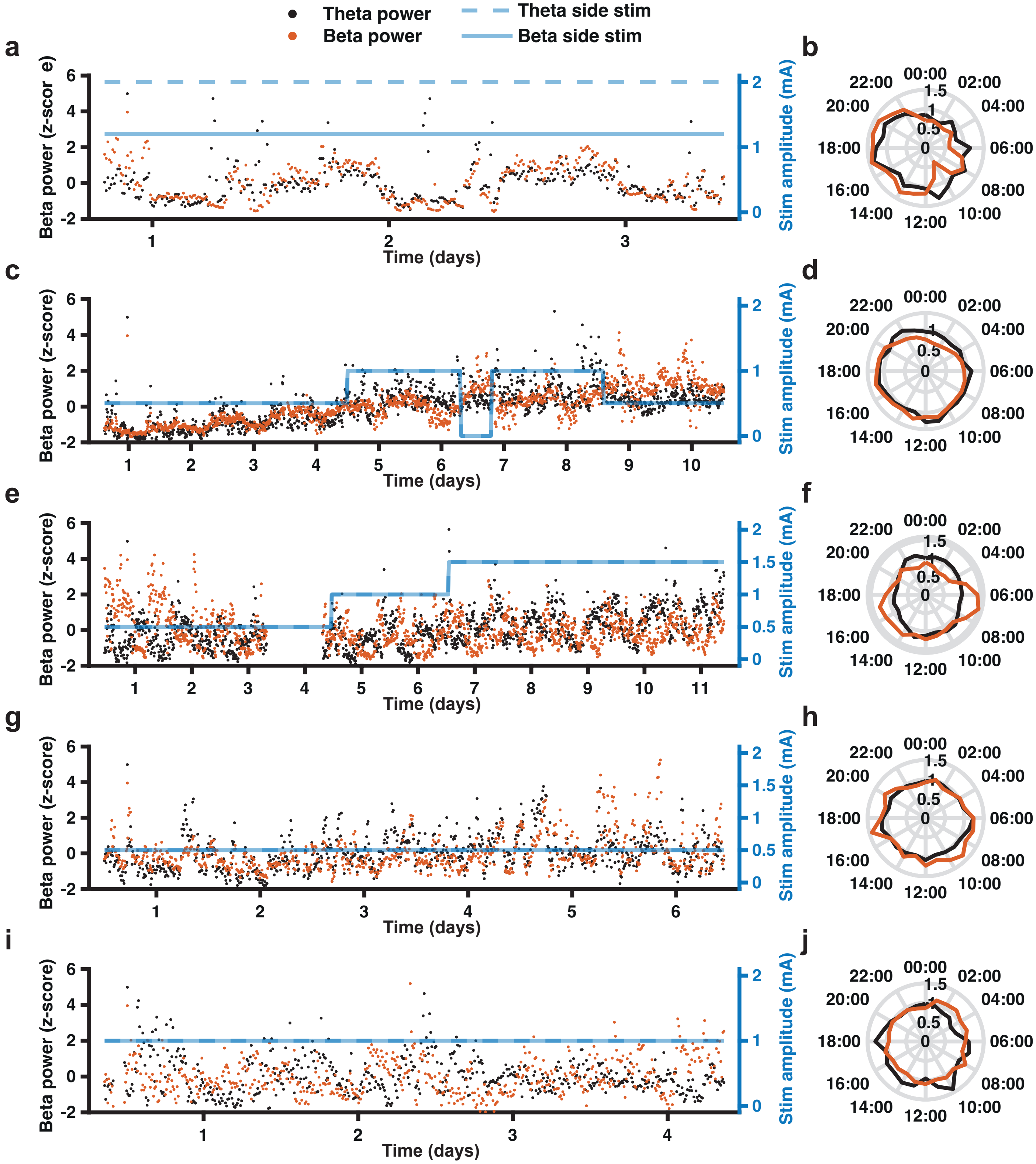

Supplementary Figure 7: Individual beta/theta and stimulation time series for patients 7-11
a,c,e,g,i: Percept measures of beta power (orange) and theta power (black) from contralateral STN leads for patients 7-11 presented as z-scores, with stimulation amplitude timeline plotted in blue (opposite y-axis; solid line = ipsilateral to beta power stream, dashed line: ipsilateral to theta power stream). **b,d,f,h,j:** Median beta (orange) and theta (black) power across the 24h diurnal cycle for patients 7-11.
